# Effect of Potent Ethyl Acetate Fraction of *Stereospermum suaveolens* Extract in Streptozotocin-Induced Diabetic Rats

**DOI:** 10.1100/2012/413196

**Published:** 2012-04-19

**Authors:** T. Balasubramanian, Tapan Kumar Chatterjee, G. P. Senthilkumar, Tamizh Mani

**Affiliations:** ^1^Department of Pharmaceutical Chemistry, Bharathi College of Pharmacy, Bharathi Nagara, Mandya, Karnataka-571 422, India; ^2^Division of Pharmacology, Department of Pharmaceutical Technology, Jadavpur University, Kolkata-700 032, India

## Abstract

To evaluate the antihyperglycemic effect of ethyl acetate fraction of ethanol extract of *Stereospermum suaveolens* in streptozotocin-(STZ-) induced diabetic rats by acute and subacute models. In this paper, various fractions of ethanol extract of *Stereospermum suaveolens* were prepared and their effects on blood glucose levels in STZ-induced diabetic rats were studied after a single oral administration (200 mg/kg). Administration of the ethyl acetate fraction at 200 mg/kg once daily for 14 days to STZ-induced diabetic rats was also carried out. The parameters such as the fasting blood glucose, hepatic glycogen content, and pancreatic antioxidant levels were monitored. In the acute study, the ethyl acetate fraction is the most potent in reducing the fasting serum glucose levels of the STZ-induced diabetic rats. The 14-day repeated oral administration of the ethyl acetate fraction significantly reduced the fasting blood glucose and pancreatic TBARS level and significantly increased the liver glycogen, pancreatic superoxide dismutase, and catalase activities as well as reduced glutathione levels. The histopathological studies during the subacute treatment have been shown to ameliorate the STZ-induced histological damage of pancreas. This paper concludes that the ethyl acetate fraction from ethanol extract of *Stereospermum suaveolens* possesses potent antihyperglycemic and antioxidant properties, thereby substantiating the use of plant in the indigenous system of medicine.

## 1. Introduction

Diabetes mellitus is a chronic disorder in the metabolism of carbohydrates, proteins, and fat due to absolute or relative deficiency of insulin secretion with/without varying the degree of insulin resistance [1]. In the recent past, many antidiabetic agents are introduced, still the diabetes and the related complications continue to be a major medical problem, not only in developed countries but also in developing countries. Medicinal plants used to treat hyperglycemic conditions are of considerable interest for ethnobotanical community as they are recognized to contain valuable medicinal properties in different parts of the plant, and a number of plants have shown varying degree of hypoglycemic and antihyperglycemic activity [2].

*Stereospermum suaveolens * (Roxb.) DC, family Bignoniaceae, commonly known as Padiri, is a large deciduous tree found throughout the moist parts of India. Different parts of the plant have been used in Ayurvedic medicine to treat various kinds of diseases. Traditionally, the decoction of bark and root is used for the treatment of pain, fever, inflammations, asthma, liver disorders, and as a diuretic [3, 4]. The flowers are mixed with honey and given orally, for the control of hiccup [5]. In southern India, the bark is used traditionally for the treatment of diabetes [3]. The root extract is known to possess anticancer activity [3, 6]. The previous phytochemical studies reported the presence of lapachol [7], sterekunthal B, stereochenols A and B [8, 9] in the bark, and scutellarein [10], stereolensin, dinatin (4,5,7-trihydroxyl-6-methoxyflavon), and dinatin-7-glucuroniside [11] in the leaves.

 Earlier studies in our laboratory have reported the antihyperglycemic and antioxidant activities of the crude ethanol extract of *Stereospermum suaveolens * (EESS) bark in experimental rats [12]. However, the antihyperglycemic activity of the fractions of this plant has not been elucidated yet, and there is a need to isolate the potent antidiabetic fraction from ethanol extract of *Stereospermum suaveolens * to obtain novel prototypes to manage diabetes mellitus. Hence, in the present work, we made an attempt to evaluate the antihyperglycemic in addition to pancreatic antioxidant effects of ethyl acetate fraction of EESS in streptozotocin-induced diabetic rats by acute and subacute models.

## 2. Materials and Methods

### 2.1. Chemicals

Streptozotocin and other chemicals were purchased from SISCO Research Laboratory, India. Glibenclamide was obtained from Prudence Pharma Chem, Ankeshwara, Gujarat. The used solvents and chemicals were analytical grade.

### 2.2. Plant Material

The plant was identified and authenticated by the Tropical Botanical Garden and Research Institute, Palode, Thiruvananthapuram district, Kerala, India and a voucher specimen (TBS-1) has been deposited in our laboratory for further reference. The bark of *Stereospermum suaveolens * (Roxb.) DC was collected during October 2006 from Palode forest, Thiruvananthapuram district, Kerala, India. The bark of the plant was dried under shade and powdered with a mechanical grinder. The powdered plant material was then passed though sieve no. 40 and stored in an air tight container for future use. 

### 2.3. Preparation of the Crude Plant Extract and Fractions

The shade dried coarse powder bark of *Stereospermum suaveolens * (500 g) was packed in the soxhlet extraction apparatus and extracted with 1.5 litre of 95% ethanol at temperature of 40–50°C for 72 h. The extract was filtered and then concentrated to dryness in a rotary evaporator under reduced pressure at temperature of 40°C. Then the crude ethanol extract of *Stereospermum suaveolens * (100 g) was dissolved in distilled water (500 mL) and fractionated with pet ether, chloroform, and ethyl acetate.

The resultant black color residues were stored in a dessicator for use in subsequent experiments and considered as the crude ethanol extract and fractions. The yield of the crude ethanol extract, pet ether, chloroform, ethyl acetate, and aqueous fractions were 25, 10, 7.16, 16.52, and 20.45% w/w, respectively. Weighed amount of fractions were suspended in 5% DMSO in normal saline prior to oral administration.

### 2.4. Animals

Male Wistar albino rats (weighing 150–200 g) and male Swiss albino mice (20–25 g) were purchased from M/S-Ghosh Enterprises, Kolkata, India. The animals were randomly grouped (*n* = 6) and housed in polyacrylic cages (38 × 23 × 10 cm) and maintained under standard laboratory conditions (25 ± 2°C) with dark and light cycle (14/10 h). They were allowed free access to standard dry pellet diet (Hindustan Lever, Kolkata, India) and water *adlibitum*. The rats were acclimatized to laboratory condition for 1 week before commencement of experiment. Ethical clearance was obtained from Jadavpur University Animals Ethical Committee for using animals in the present study.

### 2.5. Acute Oral Toxicity Study

An acute oral toxicity study was performed as per OECD-423 guidelines [13]. Male Swiss albino mice (20–25 g) were randomly distributed to twenty-four groups of three each. The animals were fasted overnight, and the fractions were administered orally at doses of 100, 200, 400, 800, 1600, and 3200 mg/kg body weight. The animals were closely observed for the first 24 h for any toxic symptoms and for 72 h for any mortality.

## 3. Experimental Design

### 3.1. Induction of Experimental Diabetes

Rats were fasted for 16 h before the induction of diabetes with STZ. A freshly prepared solution of STZ (50 mg/kg) in 0.1 M cold citrate buffer, pH: 4.5, were injected intraperitoneally in a volume of 1 mL/kg [14], and the control rats were injected with citrate buffer alone. In order to control the hypoglycemia during the first day after the STZ administration, diabetic rats were given 5% glucose solution orally. Hyperglycemia was confirmed by the elevated fasting glucose levels in blood, determined at 48 h and then on day 6 after injection. Rats with diabetes exhibiting fasting blood glucose levels in the range of 260–325 mg/100 mL were selected for the studies, and blood glucose levels were measured by reflective glucometer (Accu-chek) using the glucose oxidase method.

### 3.2. Acute Antihyperglycemic Study [15]

The rats were fasted for 16 h, divided into seven groups of six each, and treated as follows: Group I, nondiabetic control, was given 5% DMSO in normal saline orally at a dose of 5 mL/kg. Group II, STZ-diabetic control, received 5% DMSO in normal saline (5 mL/kg) orally. Groups III–VI, STZ-diabetic rats, were treated with pet ether, chloroform, ethyl acetate, and aqueous fractions (200 mg/kg, orally), respectively. Group VII, STZ-diabetic rats, was administered with standard drug Glibenclamide at a dose of 0.5 mg/kg orally. Blood samples were taken from the tail vein at 0, 0.5, 1, 2, 4, and 5 h after the oral administration, and fasting blood glucose levels were determined.

### 3.3. Subacute Antihyperglycemic Study (14 Days)

Rats were fasted for 16 h and divided into four groups of six each [16]. Group I, nondiabetic control, were given 5% DMSO in normal saline orally at a dose of 5 mL/kg. Group II, STZ-diabetic control, received 5% DMSO in normal saline at a dose of 5 mL/kg orally. Group III, STZ-diabetic rats, was treated with ethyl acetate fraction orally at a dose of 200 mg/kg. Group IV STZ-diabetic rats were administered with standard drug Glibenclamide at a dose of 0.5 mg/kg orally. The treatment was continued once daily for 14 days. Fasting blood glucose level of each animal was determined on days 1, 4, 7, 10, and 15 after the initiation of treatment. The body weights of animals were also monitored on the same days. On the 15th day, all the rats were sacrificed by euthanasia and the liver and pancreas were excised immediately and washed with ice cold saline solution.

### 3.4. Estimation of Liver Glycogen Content

The glycogen content was determined from the liver expressed as mg/g of liver tissue [17].

### 3.5. Estimation of Antioxidant Assays

After the determination of blood glucose level, the rats were sacrificed and pancreas was excised, rinsed in ice-cold normal saline (pH: 7.4), blotted dry and weighed. A 10% w/v of homogenate was prepared in 1.15% KCl and processed for the estimation of lipid peroxidation [18], reduced glutathione content (GSH) [19] (Ellman, 1959), superoxide dismutase (SOD) [20], catalase (CAT) [21], and total proteins [22].

### 3.6. Histopathological Study

The rats were sacrificed on 15th day after the determination of fasting blood glucose level, and the pancreas tissues were harvested. The fragments from the tissues were fixed in 10% neutral formalin solution, embedded in paraffin, and then, stained with hematoxylin (H) and eosin (E).

### 3.7. Statistical Analysis

The experimental data were expressed as mean ± SEM. The data were analyzed using ANOVA and Dunett's test. The results were considered statistically significant if *P* < 0.05.

## 4. Results

### 4.1. Acute Oral Toxicity Study

The pet ether, chloroform, ethyl acetate, and aqueous fractions of ethanol extract of *Stereospermum suaveolens * (EESS) did not show any mortality and toxic manifestations upto the dose of 3200 mg/kg. b.w. Further dosing was not performed to estimate the LD_50_ (lethal dose) value. According to the OECD guidelines for the acute toxicity, an LD_50_ dose of 2000 mg/kg and above is categorized as unclassified and hence the drug is found to be safe. Based on the acute toxicity studies, the dose 200 mg/kg of the fractions has been selected as the therapeutic dose.

### 4.2. Acute Antihyperglycemic Study

The effects of the pet ether, chloroform, ethyl acetate, and aqueous fractions of EESS on the blood glucose in STZ-induced diabetic rats are depicted in Table 1. The blood glucose levels were significantly (*P* < 0.001) elevated in diabetic control rats as compared to nondiabetic control rats. Oral acute administration of pet ether (*P* < 0.05, *P* < 0.001) and ethyl acetate (*P* < 0.001) fractions of EESS at the dose of 200 mg/kg significantly lowered the elevated blood glucose level in STZ-induced diabetic rats as compared to diabetic control rats, while chloroform and aqueous fractions were devoid of antihyperglycemic activity. The ethyl acetate fraction produced more potent effects than pet ether fraction in acute antihyperglycemic model.

### 4.3. Subacute Antihyperglycemic Study

#### 4.3.1. Effect on Blood Glucose Levels

The effect of the ethyl acetate fraction of EESS on the fasting blood glucose levels in STZ-induced diabetic rats is shown in Table 2. Treatment of normal control rats with vehicle (5% DMSO) alone does not affect the normal glucose concentration throughout the study. Repeated oral administration with a dose of 200 mg/kg of the ethyl acetate fraction of EESS to STZ-induced diabetic rats for 14 days significantly (*P* < 0.001) reduced the elevated fasting blood glucose levels at days 1, 4, 7, 10, and 15 after initiation of treatment, when compared to diabetic control rats. The effect of ethyl acetate fraction is comparable to that of glibenclamide.

#### 4.3.2. Effect of Fractions on Body Weight

The effect of the ethyl acetate fraction on body weight in the STZ-induced diabetic rats is given in Table 3. The body weight was slightly increased in the normal control rats (in 14 days period) compared to initial body weight, whereas in the diabetic control rats, there was a significant decrease in the body weight over the same period. The ethyl acetate fraction of EESS-treated diabetic rats gained significant weight but the increase remained lesser than the nondiabetic controls. Glibenclamide (0.5 mg/kg) treatment significantly reduced the body weight of diabetic rats.

#### 4.3.3. Effect of Hepatic Glycogen Content

The liver glycogen content was significantly decreased in STZ-induced diabetic rats as compared to nondiabetic rats, and the result is shown in Table 4. Treatment with ethyl acetate fraction of EESS significantly (*P* < 0.001) increased the liver glycogen levels in STZ-induced diabetic rats.

### 4.4. Effects on Pancreatic *In Vivo* Antioxidant Activities

#### 4.4.1. Lipid Peroxidation

STZ-induced diabetic control rats show an increased TBARS (thiobarbituric acid reactive substances) level in pancreas (Table 4) as compared to nondiabetic rats. However, ethyl acetate fraction was significantly (*P* < 0.001) decreased TBARS as compared to respective diabetic control rats.

#### 4.4.2. Reduced Glutathione Content

The total GSH content decreased in STZ-induced diabetic rats as compared to nondiabetic rats. Treatment with ethyl acetate fraction were significantly (*P* < 0.001) increased total GSH in pancreas when compared to STZ-induced diabetic control rats and is shown in Table 4.

#### 4.4.3. Superoxide Dismutase and Catalase

SOD and catalase (CAT) activities in the STZ-induced diabetic rats were significantly (*P* < 0.001) decreased in pancreas. Administration of ethyl acetate fraction significantly (*P* < 0.001) increased the SOD and CAT activities compared to STZ-induced diabetic control rats and is shown in Table 4.

#### 4.4.4. Histopathological Studies of Pancreas

**Figure 1:**
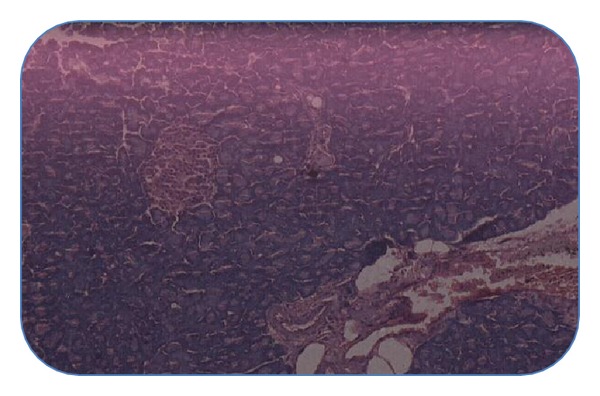
Pancreatic section of normal rat stained with haemotoxylin and eosin.

**Figure 2:**
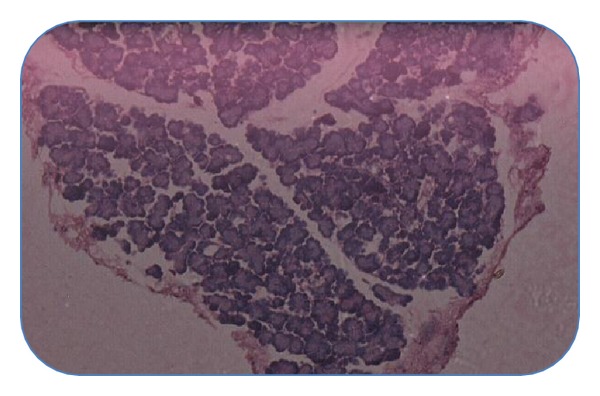
Pancreatic section of STZ-intoxicated rat stained with haemotoxylin and eosin.

**Figure 3:**
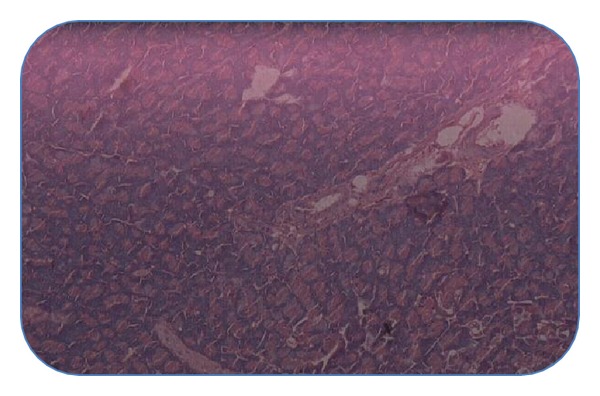
Pancreatic section of ethyl acetate fraction + STZ-treated rat stained with haemotoxylin and eosin.

**Figure 4:**
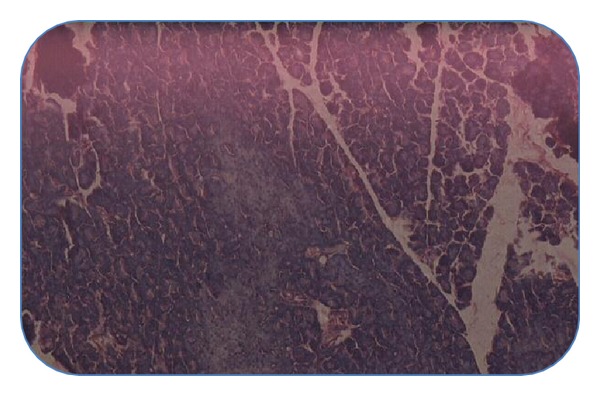
Pancreatic section of Glibenclamide + STZ-treated rat stained with haemotoxylin and eosin.

Histopathological section of nondiabetic control pancreas (Figure 1) showing normal islets with clusters of purple-stained *β*-cells.  Figure 2 presents the section of diabetic pancreas showing atrophy of *β*-cells and vacuolar degenerative changes in islets, and mild infiltration of inflammatory cells. It is characterized by a reduction in number and size of islets. Figures 3 and 4, treatment with ethyl acetate fraction and glibenclamide, show maximum cellular regeneration of pancreatic *β*-cells with well-granulated and an increased number of islets.

## 5. Discussion

The present study was undertaken to find out the potent antihyperglycemic fraction from ethanol extract of *Stereospermum suaveolens * (EESS). Diabetes mellitus affects both glucose and lipid metabolism [23]. In diabetes, the increased blood sugar levels might be due to either insulin resistance of the body cells or decreased secretion of insulin from **β**-cells manifested in the decreased serum insulin levels. The fundamental mechanism underlying hyperglycemia in diabetes mellitus involves the overproduction of glucose by excessive hepatic glycogenolysis and gluconeogenesis, decreased hepatic glycogenesis and, or decreased utilization of glucose by the tissue [24].

Experimental diabetes mellitus was induced by injecting STZ, which is probably due to the destruction of **β**-cells of islets of langerhans [25, 26] of pancreas leading to high levels of blood glucose in rats. As a preliminary antihyperglycermic activity assessment as well as to isolate a potent fraction, the various fractions of EESS were administrated to STZ-induced diabetic rats at a single dose level (200 mg/kg) to determine the acute effect on blood glucose concentration. Consequently, the pet ether and ethyl acetate fractions showed significant antihyperglycemic activity in STZ-induced diabetic rats, while no remarkable effect on blood glucose level was observed on the rats treated with chloroform and aqueous fractions. However, in comparison to pet ether fraction, ethyl acetate fraction has shown potential activity in decreasing the blood glucose level in STZ-induced diabetic rats.

In subacute study, daily administration of ethyl acetate fraction of EESS for 14 days significantly decreased the blood glucose levels in STZ-induced diabetic rats, when compared to the diabetic control rats. This data support our previous investigation [12] in STZ diabetic rats where EESS significantly reduced fasting blood glucose at the end of 14-day period. The possible mechanism of the ethyl acetate fraction for its antihyperglycemic effect may be through potentiation of pancreatic secretion of insulin from remaining *β*-cells of islets and/or regenerated **β**-cells or due to enhanced transport of blood glucose to the peripheral tissues and/or the reduction of hepatic gluconeogenesis and glycogenolysis, and increased hepatic glyconeogenesis. The histopathological studies of pancreas also show that the ethyl acetate fraction of EESS regenerates the **β**-cells. This finding suggests that the antihyperglycemic activity of ethyl acetate fraction of EESS may be due to potentiation of insulin secretion by regeneration of **β**-cells.

STZ-induced diabetes is characterized by severe loss in body weight. This may be due to increased muscle wasting and due to loss of tissue proteins [27]. In this study, a significant weight loss was observed in the STZ-induced diabetic control rats. The ethyl acetate fraction treated rats showed significant recovery in body weight gain when compared to diabetic control rats. This may be due to controlling muscle wasting and improvement in insulin secretion as well as glycemic control by the ethyl acetate fraction.

Glycogen is the primary intracellular storage form of glucose and its levels in various tissues especially liver and skeletal muscle are a direct reflection of insulin activity as insulin promotes intracellular glycogen deposition by stimulating glycogen synthase and inhibiting glycogen phosphorylase [28, 29]. The decrease in glycogen content of liver in STZ-induced diabetic rats observed in the present study is probably due to the lack of or resistance to insulin, which is essential to stimulate glycogenesis and/or inhibit glycogenolysis [30, 31]. The significant increase of liver glycogen levels in the ethyl acetate fraction treated rats may be due to stimulation of glyconeogenesis and/or inhibition of glycogenolysis in liver.

Oxidative stress, a key pathogenic factor in the development of diabetic complications, induces the production of highly reactive oxygen species that are toxic to the cell membrane in which these radicals interact with the lipid bilayer and produce lipid peroxides [32–34].

The TBARS levels were measured in pancreas and its levels found to be significantly (*P* < 0.001) increased in diabetic control rats. In the present study, TBARS levels in pancreas were significantly lower in the ethyl acetate fractions treated rats compared to the diabetic control group. This suggests that ethyl acetate fraction of EESS may protect the pancreas tissues from lipid peroxidation.

GSH is the major endogenous antioxidant that counters balance free-radical-mediated damage in diabetes mellitus [35]. It is well known that GSH is involved in the protection of normal cell structure and function by maintaining the redox homeostasis, quenching of free radicals and by participating in detoxification reactions. The GSH levels were significantly (*P* < 0.001) decreased in pancreas of STZ-induced diabetic rats. The decrease in GSH levels represents increased utilization due to oxidative stress induced by STZ [36]. The increased GSH content in pancreas of the rats treated with ethyl acetate fraction of EESS may be a factor responsible for inhibition of lipid peroxidation. Hence, the elevated level of GSH protects cellular proteins against oxidation through glutathione redox cycle and also directly detoxifies reactive oxygen species generated from exposure to STZ [37].

The present study also revealed that SOD and CAT levels were decreased in pancreas in diabetic rats. SOD is an important defense enzyme, which catalyses the dismutation of superoxide radicals. CAT is a hemeprotein that catalyses the reduction of hydrogen peroxides and protects the tissues from highly reactive hydroxyl radicals [38]. Therefore, reduction in the activity of these enzymes (SOD, CAT) may result in a number of deleterious effects due to the accumulation of superoxide anion radicals and hydrogen peroxide [39]. These antioxidant enzymes SOD and CAT levels significantly increased after the treatment of ethyl acetate fraction of EESS in STZ-induced diabetic rats indicating the free radical scavenging activity and their protective effect against cellular damage. Antioxidants may have a role in the prevention of diabetes. The above *in vivo *antioxidant status reveals support to antidiabetic effect of ethyl acetate fractions.

## 6. Conclusion

The present research clearly indicates that the ethyl acetate fraction of ethanol extract of *Stereospermum suaveolens * exhibits antihyperglycemic in addition to antioxidant effects in STZ-induced diabetic rats, thereby justifying its ethnomedicinal use. Further chemical and pharmacological investigations are in progress to elucidate in detail the active principles and mechanism of action of ethyl acetate fraction of EESS.

## Figures and Tables

**Table 1:** Effect of various fraction of ethanol extract of *Stereospermum suaveolens* on glucose level by acute treatment in STZ-induced diabetic rat.

Groups	0 h	1/2 h	1 h	2 h	4 h	5 h
Normal (5% DMSO in 0.9% NaCl, 5 mL /kg )	84.16 ± 2.34	85.00 ± 2.68	87.73 ± 2.21	90.23 ± 2.05	89.13 ± 2.34	83.83 ± 2.44
STZ-induced diabetic control (40 mg/kg)	294.65 ± 1.86 ^a,∗∗^	300.21 ± 3.57^a,∗∗^	302.25 ± 6.10^a,∗∗^	298.16 ± 5.31^a,∗∗^	299.56 ± 6.24^a,∗∗^	305.16 ± 4.82^a,∗∗^
STZ+ Pet ether fraction (200 mg/kg)	284.04 ± 1.82	280.14 ± 5.76	265.16 ± 6.28^b,∗^	256.53 ± 6.72^b,∗∗^	248.83 ± 6.42^b,∗^	234.71 ± 5.67^b,∗∗^
STZ + Chloroform fraction (200 mg/kg)	267.24 ± 1.46	262.63 ± 1.45	258.84 ± 2.18	254.5 ± 2.14	253.49 ± 2.81	261.24 ± 3.81
STZ + Ethyl acetate fraction (200 mg/kg)	264.81 ± 1.96	241.16 ± 4.54	229.23 ± 6.14^b,∗∗^	212.38 ± 6.51^b,∗∗^	197.67 ± 7.68^b,∗∗^	187.28 ± 6.436^b,∗∗^
STZ + Aqueous fraction (200 mg/kg)	286.90 ± 2.31	285.09 ± 3.81	281.84 ± 2.18	279.30 ± 2.72	272.65 ± 2.73	279.50 ± 3.73
STZ + Glibenclamide (0.5 mg/kg)	286.50 ± 6.35	255.50 ± 8.08^b,∗∗^	224.83 ± 7.18^b,∗∗^	162.00 ± 7.81^b,∗∗^	162.00 ± 7.81^b,∗∗^	111.50 ± 7.13^b,∗∗^

Values are given as mean ± SEM, 6 rats in each group,

^a, ∗∗^ *P* < 0.001 as compared to normal control group,

^b, ∗^ *P* < 0.05, **P < 0.001, when compared with STZ-treated control group.

**Table 2:** Effect of ethyl acetate fraction of *stereospermum suaveolens* on glucose level in STZ-induced diabetic rats.

Groups	Serum glucose levels (mg/dL)				
	1 day	5th day	7th day	10th day	15th day
Normal (5% DMSO in 0.9% NaCl, 5 mL/kg )	85.64 ± 1.99	85.84 ± 2.18	87.84 ± 2.18	91.64 ± 1.36	86.60 ± 1.91
STZ-induced diabetic control (40 mg/kg)	290.65 ± 1.86^a,#^	294.80 ± 2.35^a,#^	294.84 ± 2.18^a,#^	299.2 ± 1.46^a,#^	293.40 ± 2.42^a,#^
STZ + Ethyl acetate fraction (200 mg/kg)	264.81 ± 1.96^b,∗^	247.86 ± 1.86^b,∗∗^	211.84 ± 2.18 ^b,∗∗^	187.6 ± 2.18^b,∗∗^	129.42 ± 1.9^b,∗∗^
STZ+ Glibenclamide (0.5 mg/kg)	286.50 ± 6.35	173.50 ± 6.35^b,∗^	141.72 ± 6.26^b,∗^	126.21 ± 6.61^b,∗^	115 ± 6.11^b,∗^

Values are given as mean ± SEM, 6 rats in each group,

^a, #^ *P* < 0.001 as compared to normal control group,

^b, ∗^ *P* < 0.05, **P < 0.001, when compared with STZ-treated control group.

**Table 3:** Effect of ethyl acetate fraction of *Stereospermum suaveolens* on body weight in STZ-induced diabetic rats.

Groups	Body weight (g)				
	1st day	4th day	7th day	10th day	15th day
Normal (5% DMSO in 0.9% NaCl, 5 mL/kg)	185.73 ± 1.96	185.86 ± 1.32	196.00 ± 3.37	193.72 ± 3.24	197.26 ± 2.06
STZ-induced diabetic control (40 mg/kg)	181.25 ± 2.61	171.63 ± 2.70^a, #^	164.81 ± 3.73^a, #^	154.53 ± 2.74^a, #^	154.13 ± 1.86^a,#^
STZ + Ethyl acetate fraction (200 mg/kg)	182.60 ± 3.24	184.57 ± 0.64^b,∗∗^	189.12 ± 2.38^b,∗∗^	191.62 ± 2.29^b,∗∗^	194.57 ± 1.25^b,∗∗^
STZ + Glibenclamide (0.5 mg/kg)	184.06 ± 3.22	172.50 ± 0.84	169.13 ± 2.40	164.26 ± 2.21	159.56 ± 1.45

Values are mean ± SEM, 6 rats in each group,

^a, #^ *P* < 0.001 as compared to normal control group,

^b, ∗∗^ *P* < 0.001, when compared with STZ-treated control group.

**Table 4:** Effect of ethyl acetate fraction of *Stereospermum suaveolens* on pancreatic lipid peroxidation, glutathione, and antioxidants in STZ-induced diabetic rats.

Groups	Lipid peroxidation (nmol of MDA/mg protein)	Glutathione (*μ*M/gm protein)	Superoxide dismutase (IU/mg protein)	Catalase (nmol of H_2_O_2_ decomposed/min/mg protein)	Liver glycogen (mg/gm of liver)
Normal (5% DMSO in 0.9% NaCl, 5 mL/kg)	11.13 ± 0.56	49.28 ± 1.26	26.42 ± 0.68	56.26 ± 1.82	35.20 ± 0.89
STZ-induced diabetic control (40 mg/kg)	35.46 ± 0.43^a,#^	23.58 ± 1.79^a,#^	9.63 ± 0.67^a,#^	28.45 ± 1.48^a,#^	12.31 ± 0.63^a,#^
STZ + Ethyl acetate fraction (200 mg/kg)	14.37 ± 0.56^b, ∗∗^	44.38 ± 1.24^b, ∗∗^	20.31 ± 0.42^b,∗∗^	51.72 ± 1.26^b, ∗∗^	29.84 ± 0.49^b,∗^
STZ + Glibenclamide (0.5 mg/kg)	22.24 ± 0.62	37.56 ± 1.36	16.46 ± 0.67	43.21 ± 1.42	24.42 ± 1.40^b,∗^

Values are mean ± SEM, 6 rats in each group,

^a, #^ *P* < 0.001 as compared to normal control group,

^b, ∗^ *P* < 0.05, **P < 0.001, when compared with STZ-treated control group.
